# The Power of Faith: The Influence of Athletes’ Coping Self-Efficacy on the Cognitive Processing of Psychological Stress

**DOI:** 10.3389/fpsyg.2019.01565

**Published:** 2019-07-11

**Authors:** Tengfei Guo, Yakun Ni, Qiaoling Li, Hao Hong

**Affiliations:** ^1^ School of Educational Science, Henan University, Kaifeng, China; ^2^ School of Physical Education, Henan University, Kaifeng, China

**Keywords:** coping self-efficacy, psychological stress, N1, FRN, P300, athletes

## Abstract

Coping self-efficacy (CSE) has a positive mental health effect on athletes’ ability to cope with stress. To understand the mechanism underlying the potential impact of CSE, event-related potentials (ERPs) were used to explore the neural activity of the cerebral cortex under acute psychological stress in athletes with different CSE levels. Among 106 high-level athletes, 21 high-CSE athletes and 20 low-CSE athletes were selected to participate in the experiment. A mental arithmetic task was used to induce acute psychological stress. The results showed that high-CSE athletes responded more quickly than low-CSE athletes. In the stress response stage, the N1 peak latency of low-CSE athletes was longer than that of high-CSE athletes, and the N1 amplitude was significantly larger than that of high-CSE athletes. In the feedback stage, the FRN amplitude with error feedback of high-CSE athletes was larger than that of low-CSE athletes, and the P300 amplitude with correct feedback was larger than that with error feedback. The results indicate that high-CSE athletes can better cope with stressful events, adjust their behaviors in a timely manner according to the results of their coping, and focus more on processing positive information.

## Introduction

As a high-stress group expected to perform in an intensely competitive environment, athletes face various stressful events. Factors such as the time pressures of the game, noise from the audience, and the uncertainty of competition, all place athletes in a state of high tension, cause acute psychological stress. Unlike the physiological stress caused by situations such as pain and hunger, psychosocial stress is mainly induced by socially threatening situations such as social evaluation, social exclusion, and achievement/cognitive stress and occurs when an individual’s psychological homeostatic process is threatened (Pruessner et al., 2010; Kogler et al., 2015). Studies show that stress is the main cause of athletes’ mental health problems (Gulliver et al., 2015; Rice et al., 2016; Sabato et al., 2016; Gerber et al., 2018). Many athletes cannot withstand psychological pressure before and during competition, thus affecting their physical and technical performance and eventually preventing them from achieving the desired results (Moritz et al., 2000; Nicholls et al., 2010). Therefore, it is particularly important for athletes to be able to cope with the pressures of competition.

Ideal athletic performance occurs when an athlete successfully copes with various adverse situations during competition. The pressure cognitive interaction theory argues that coping is an important regulatory variable of the psychosocial stress that affects individuals’ physical and mental health (Lazarus and Folkman, 1984; Edwards and Cooper, 1988). As a pressure buffer and resource replenishment device, coping helps athletes self-regulate and eliminate the interference caused by stress and helps them quickly adapt to stressful situations that arise in competition (Nicholls and Perry, 2016). When facing a stressful situation, the more that an athlete can mobilize resources such as cognitive level, self-confidence, experience, and willpower, the higher the motivation level is, and the more active the involvement is. Bandura (1997) proposes that individuals’ motivation level, emotional state, and behavior are based more on what they believe than on what is objective and true. Coping self-efficacy (CSE) is an extension of self-efficacy theory in the field of coping and refers to an individual’s confidence about his/her ability to cope successfully with stress (Benight et al., 1997). CSE is considered an important influencing factor in athletes’ ability to effectively cope with the stress of competition (Gyurcsik et al., 2010).

As an intrinsic and relatively stable individual belief, CSE directly affects an athlete’s ability to cope effectively with stress. Therefore, exploring the mechanism of CSE as a potential belief helps illuminate why some athletes feel more confident than others about their ability to cope effectively with stress. This study uses the event-related potential (ERP) technique to reveal the cortical neurological activity induced by acute psychological stress in athletes with different CSE levels. The brain is believed to be the organ that plays a core role in stress reactivity, coping, and recovery processes (McEwen, 2009; McEwen and Gianaros, 2010). In response to stress, the brain activates several neuropeptide-secreting systems. The brain first processes various stimuli deemed threats and then induces endocrine responses *via* the hypothalamus-pituitary-adrenal axis (HPA axis) and the sympathetic adrenal medulla axis (De Kloet et al., 2005; Foley and Kirschbaum, 2011), which in turn generate physiological and behavioral responses to the stimuli. Therefore, attention-related brain cognitive processes are particularly important in competitive sports. Individuals’ information processing ability is limited. Therefore, in fast ball sports such as basketball, athletes must select prominent key information for processing from a large corpus of information (Isoglualkac et al., 2018). Using the ERP technique in a high temporal resolution enables understanding the intracerebral temporal dynamic changes during the attention-processing process of athletes under stress.

Uncontrollability and social-evaluated threats are the two key stressors responsible for acute psychological stress (Dickerson and Kemeny, 2004). Based on previous studies (Yang et al., 2012; Qi et al., 2018), this study used the multiplication estimation task and designed an experimental situation to induce the psychological stress response of participants by limiting the time available for decision-making (to induce uncontrollability) and informing the participant that his/her correct answer rate would be compared with that of another participant, and a reward would be given accordingly (to induce social-evaluated threat). The study by Qi et al. (2016) showed that the individual’s salivary cortisol content significantly increased after a mental arithmetic task, confirming the task’s successful induction of acute psychological stress response. When stressed, high-CSE individuals are more confident about their ability to face the challenges of stress and adopt effective coping strategies to maintain their physical and mental health. In contrast, low-CSE individuals have insufficient self-confidence and cannot effectively or timely relieve various psychological and physical symptoms caused by stress, resulting in threats to their health (Watson and Watson, 2016). To understand the potential influencing factors of athletes’ CSE and improve the ability of high-level athletes to cope with stress, this study used the ERP technique to explore differences in the neurological activities of athletes with different CSE levels under psychological stress and stress assessment feedback, thereby further illuminating the mechanism underlying the effect of CSE on individuals under stress.

Acute psychological stress causes the body to be in a state of high vigilance and high arousal, making early sensory coding sensitive (Löw et al., 2015). If the stimulus requires an individual to respond quickly, attention should be directed to the perception process that primarily manifests in the N1 component, and the increased vigilance should trigger a more negative N1 component (Shackman et al., 2011). Low-CSE individuals often show insufficient confidence when they are under stress, unable to effectively control stress situations, and are in a state of high tension and anxiety (Nicholls et al., 2010). Therefore, we predicted that, compared with high-CSE athletes, low-CSE athletes would show a larger amplitude of N1 under stress. Stress also has a regulatory effect on the allocation of attention resources (Shackman et al., 2011; Sänger et al., 2014; Löw et al., 2015). Some studies have shown that, stress is helpful in narrowing the focus of attention and has a negative impact on the allocation of attention resources (Dambacher and Hübner, 2015; Qi et al., 2016). As a “control switch” for allocating decision resources, the P2 component is linked to attention selection and control processing. The larger the P2 amplitude, the higher the individual’s attention level (Yuan et al., 2016). Since the high-CSE individual is more confident in accepting the tasks of stress and challenge, and the attention resources are weakened by the reduction of stress, in this sense, we predicted that the P2 amplitude of high-CSE athletes is larger than that of athletes with low CSE under stress.

“Assessment” is the core concept in the theory of stress-cognitive interaction (Lazarus and Folkman, 1984). To adapt to a changing environment, individuals must monitor the appropriateness of their current behavior and adjust their behavior accordingly. For example, after erring or receiving an error feedback, individuals must adjust their behavior to reduce the possibility of committing a similar error. In a stressful environment, the assessment of coping outcomes affects an individual’s subsequent coping efforts (Crocker et al., 2015). Previous studies showed that the result assessment process consisted of two main stages: the early stage of elementary automatic rapid assessment processing, as characterized by feedback-related negativity (FRN), and the late stage of top-down sophisticated control processing, which affected the allocation of attention resources and was characterized by P300 (Wu and Zhou, 2009; Leng and Zhou, 2010). The existing literature still lacks ERP evidence related to the assessment of stress results. This study uses FRN and P300 as indicators to investigate the temporal dynamic characteristics of the process of feedback assessment under stress. When facing the wrong response or getting the wrong feedback, they have to adjust their behavior in a timely manner to reduce the possibility of making errors. FRN reflects cognitive processing of expected error monitoring (Holroyd and Coles, 2002), an individual with high CSE can make self-evaluation of the effectiveness of responding more promptly to dangerous situations (Benight et al., 1999). In this sense, we hypothesize that in the wrong feedback phrase, the FRN amplitude of athletes with high CSE is larger than that of athletes with low CSE. In addition, previous study of the impact of stress on executive control resources (Shields et al., 2016) suggests that stress will reallocate the executive control resources from working memory and cognitive flexibility to selective attention, in order to focus on processing current stress-related information. P300, which reflects the allocation of cognitive resources in the late stage of information processing, is a neurological index of selective attention; the more the cognitive resources are occupied, the larger the P300 component is induced (Kopp and Lange, 2013). When interacting with the environment, the athletes with high CSE are inclined to positively control the environment and events that may cause stress, while the athletes with low CSE are inclined to think about personal deficiencies and take the potential difficulties more seriously than they really are (Nicholls et al., 2010). Therefore, on the basis of “mood congruent effect,” we assume that, the athletes with high CSE will have larger P300 amplitudes in positive feedback processing, while the athletes with low CSE will have a larger P300 amplitude in negative feedback processing.

## Materials and Methods

### Participants

We recruited high-level basketball players from several universities located in central part of China. The participants consisted of 106 high-level basketball players who were mostly players of Chinese University Basketball Association (CUBA). They had to pass rigorous physical and cognitive tests to become CUBA registered athletes. Before the participants registered for the experiment, the researcher explained the purpose of the project and asked them for their consent to participate. Firstly, we employed coping self-efficacy scale (Chesney et al., 2006) to test the degree of coping self-efficacy (CSE) of 106 high-level basketball players. Secondly, according to their CSE scores’ ranking, the participants whose CSE scores were ranking in top 27 percent of all participants were assigned to the high-CSE group (including 29 participants) and the participants whose CSE scores were ranking in the bottom 27 percent of all participants were assigned to the low-CSE group (including 29 participants). The CSE scores of high-CSE group ranged from 66 to 75 points (*M* = 69.21, SD = 2.93), and the CSE scores of low-CSE group ranged from 43 to 58 points (*M* = 53.97, SD = 3.86). For 1 week before the start of the experiment, all participants were allowed to refrain from taking any coffee and getting plenty of rest. Finally, 41 athletes also volunteered later to participate in the experiment, of which the high-CSE group consisted of 21 high-level basketball players (including 17 males and 4 females), and the low-CSE group consisted of 20 high-level basketball players (including 15 males and 5 females). Participants in the high-CSE group had an average age of 20.9 ± 1.34 years and included four national first-class athletes and 17 national second-class athletes, who played the forward (9), center (5), and defender (7) positions in the field. Participants in the low-CSE group had an average age of 20.7 ± 1.03 years and included two national first-class athletes and 18 national second-class athletes, who played the forward (10), center (4), and defender (6) positions in the field. The CSE score of the high-CSE group (69.52 ± 2.94) was significantly higher than that of the low-CSE group (52.80 ± 4.06), with *t*(39) = 15.16, *p* < 0.001, and Cohen’s *d* = 4.74. All participants in the experiment were right-handed, with normal naked eyesight or corrected visual acuity, and were participating in such experimental research for the first time. Before participating in the experiment, the participants were asked to refrain from taking any coffee and having a good sleep. In accordance with experimental ethical principles, all basketball players participating in the experiment signed an informed consent form prior to the experiment and received an honorarium of 40 CNY cash after completing the experiment.

### Design and Materials

The multiplication estimation task imposed a time limit for answering and included a social-evaluated threat. This task was used to induce a psychological stress response in participants (Qi et al., 2018). Two hundred and forty multiplication arithmetic problems were presented. The problems consisted of two numbers less than 10 and with two decimals (for example, 2.36 × 4.59). The numbers were selected from a series of Gaussian distributed numbers in the range of 1–10, with an average of 5 and a standard deviation of 2.5. The participants were required to determine whether the result of each arithmetic problem was less than 10 and press the “1” key if so and the “2” key otherwise. After an answer was submitted, the result was displayed as “correct,” “wrong,” or “time out.”

A single-factor inter-participant design was used. The independent variable was the type of basketball player participating in the experiment, namely, high-CSE or low-CSE basketball player. The dependent variable was the response time, the accuracy rate of the participant under stress, and the electroencephalographic (EEG) data of the participants in the stress stage and the feedback stage.

### Procedure

The experiment was conducted in a quiet and soundproof EEG laboratory. The participant wore an electrode cap, which positioned both eyes approximately 80 cm from the computer screen. Stimulating materials were presented using E-Prime 2.0 (Psychology Software Tools, Inc., Sharpsburg, USA) and appeared in black on a white background at the center of a 19-inch computer screen at a viewing angle of 6.66° × 4.87°. After the experiment started, the participants familiarized themselves with the experimental tasks and keyboard operations through the instructions and then performed the corresponding practice experiments. After they fully understood and could independently and skillfully complete the experimental procedures, the experiment formally began.

**Figure 1:**
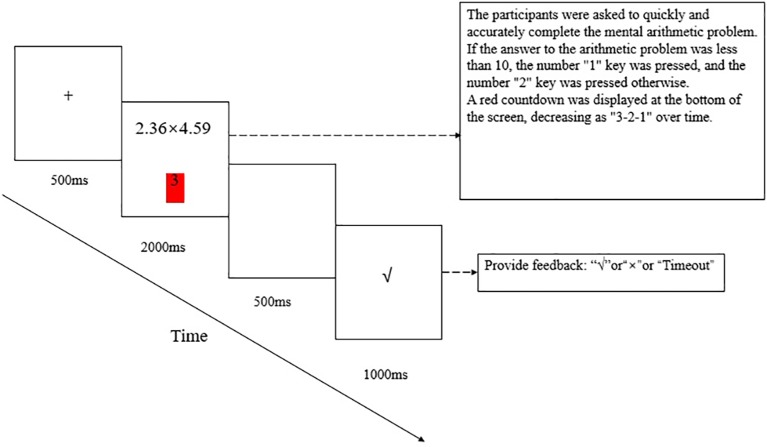
Experimental procedure and sample materials. Each trial began with a fixation cross of 500 ms, after which an arithmetic problem was displayed. The time limit for answering a problem was 2,000 ms. After the “1” or “2” key was pressed or after time expired, a blank screen appeared for 500 ms, followed by feedback for 1,000 ms and the next trial started.

At the beginning of the experiment, a “+”-shaped gaze point appeared in the center of the screen for 500 ms to remind the participants to focus on the experiment, after which an arithmetic problem was displayed. The participants were asked to quickly and accurately complete the mental arithmetic problem and submit their answer by pressing one of two keys. If the answer to the arithmetic problem was less than 10, the number “1” key was pressed, and the number “2” key was pressed otherwise. The time limit for answering a problem was 2,000 ms, and a red countdown was displayed at the bottom of the screen, decreasing as “3-2-1” over time. After the “1” or “2” key was pressed or after time expired, a blank screen appeared for 500 ms, followed by feedback, i.e., correct, wrong, and timeout, for 1,000 ms. Then, the next trial began. The experiment consisted of 240 trials, and after every 60 trials, there was a rest time controlled by the participants. The detailed experimental procedure is shown in Figure 1.

### Electrophysiological Recordings

The EEG information was recorded using a BrainAmp system with 64-channel electrodes (Brain Products, Germany) extended with the 10-20 International EEG Recording System. The vertical electrooculogram (VEOG) signal above the left eye and the horizontal electrooculogram (HEOG) signal outside the right eye were recorded with the reference electrodes at the bilateral mastoids behind the left and right ears. The ground electrode was placed at the midpoint AFz of the line connecting FPz and Fz. Before the start of the experiment, the resistance of the connecting point between each electrode and the scalp was reduced to less than 5 kΩ, the sampling frequency was set to 500 Hz, and the filter bandpass was set to 0.05–100 Hz.

### Data Analysis

To analyze the behavioral data, the participants’ reaction time and accuracy were recorded using E-prime 2.0, and the data for both variables were combined and extracted. A *t*-test was then performed using the SPSS 20.0 statistical software program.

The EEG data were processed offline using BrainVision Analyzer version 2.04 (Brain Product GmbH; Gilching, Germany). The ICA method was used to correct the ocular power. The EEGs were segmented into 1000 ms epochs surrounding the onset of the probe stimulus. The filter passband frequency was 0.01–30 Hz, and artifact signals with an amplitude greater than ±80 μV were removed. Based on the experimental design, the EEG in the stress stage was superimposed by participant type, and the EEG component in the feedback stage was superimposed and analyzed by participant type and feedback type (correct/wrong). Data with excessive artifacts and insufficient times of superimposition were eliminated. Two participants in the low-CSE group were deleted due to excessive artifacts, which resulted in EEG data that could not be superimposed or averaged. The average numbers of superimposition for the retained cases were as follows. In the high-CSE group, the number for the stress stimulus was 179.2 ± 44.9, with a range of 91–236; the number for correct feedback was 83.1 ± 32.3, with a range of 33–133; and the number for error feedback was 74.7 ± 20.2, with a range of 33–128. In the low-CSE group, the number for the stress stimulus was 175.7 ± 41.3, with a range of 78–231; the number for correct feedback was 80.9 ± 34.1, with a range of 32–146; and the number for error feedback was 70.9 ± 29.1, with a range of 30–110.

**Figure 2:**
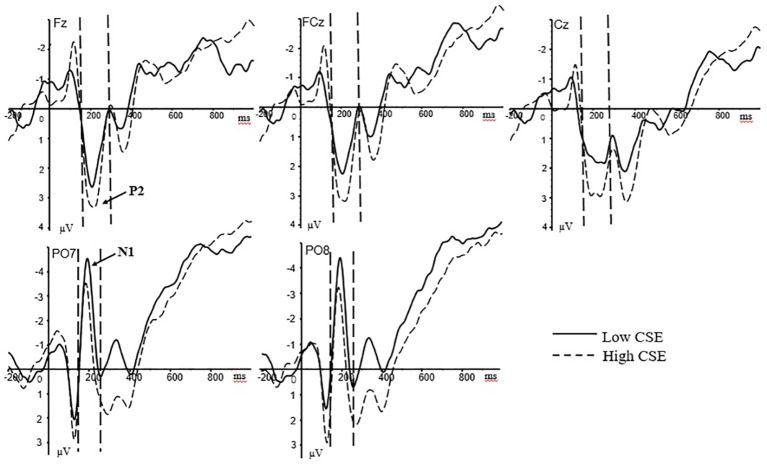
Grand-averaged ERPs at the Fz, FCz, Cz, PO7, and PO8 electrode sites for high- and low-CSE athletes under psychological stress. The P2 component (180–260 ms) at Fz, FCz, and Cz. The N1 component (150–220 ms) in PO7 and PO8.

In the stress stage, based on the total average waveform and previous studies (Yang et al., 2012; Qi et al., 2018), the N1 and P2 components were mainly analyzed. For the N1 component, the peak latency and average amplitude of the left (PO7, O1) and right (PO8, O2) sides of the cerebral palpebral area within the time window of 150–220 ms after the presentation of the stimulus were selected for analysis. For the P2 component, the average amplitudes of the frontal (F3, Fz, F4), the front-central (FC3, FCz, FC4), and the central (C3, Cz, C4) zones of the brain within the time window of 180–260 ms after the presentation of the stimulus were analyzed. N1 was subjected to a 2 (group: high/low) × 2 (hemisphere: left/right) mixed variance analysis. P2 was subjected to a 2 (group: high/low) × 3 (brain area: anterior/middle/posterior) mixed variance analysis. The grand-averaged ERPs at the Fz, FCz, Cz, PO7, and PO8 electrode sites for high- and low-CSE athletes under psychological stress stage is shown in Figure 2.

**Figure 3:**
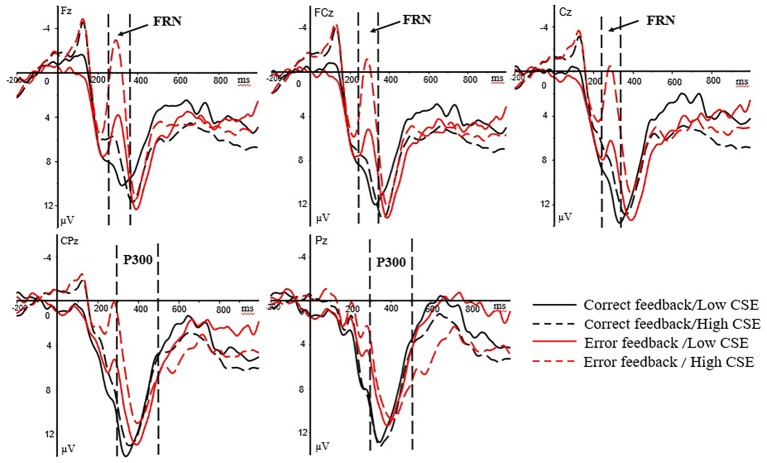
Grand-averaged ERPs at the Fz, FCz, Cz, CPz, and Pz electrode sites for high- and low-CSE athletes under the feedback. The FRN component (250–350 ms) at Fz, FCz, and Cz. The P300 component (300–500 ms) at CPz and Pz.

In the feedback stage, the ERP waveforms of the participants were analyzed for both correct and error feedback. Following previous studies on outcome evaluation (Chen et al., 2013; Ma et al., 2015), two ERP components, FRN and P300, which were related to outcome evaluation, were selected for analysis. Previous studies (Hajcak et al., 2005, 2007) showed that the maximum amplitude of FRN occurred in the anterior middle of the scalp. Therefore, the average amplitude of three electrode points at Fz, FCz, and Cz in the anterior middle of the brain at 250–350 ms after the presentation of feedback was selected for FRN analysis. The maximum amplitude of P300 appeared in the posterior of the scalp (Yeung and Sanfey, 2004). Therefore, the average amplitude of two electrode points at CPz and Pz in the posterior of the brain at 300–500 ms after the presentation of feedback was selected for P300 analysis. Because the analysis time courses for the FRN component and the P300 component partially overlapped, the FRN was defined as the most negative peak within 250–350 ms (Rigoni et al., 2010), and the FRN difference wave for error and correct feedback (dFRN, the amplitude of the brain wave caused by error feedback minus the amplitude caused by correct feedback) was analyzed (Holroyd and Krigolson, 2007). The dFRN indicator for analysis is the average amplitude of the difference wave within the time window of 250–350 ms. The FRN was subjected to a 2 (group: high/low) × 2 (feedback type: correct/error) × 3 (electrode point: Fz, FCz, Cz) mixed variance analysis, and the P300 was subjected to a 2 (group: high/low) × 2 (feedback type: correct/error) × 2 (electrode point: CPz, Pz) mixed variance analysis. The difference wave dFRN was subjected to a 2 (group: high/low) × 3 (electrode point: Fz, FCz, Cz) mixed variance analysis. When the statistical results did not pass the spherical test, the Greenhouse-Geisser method was used to correct degree of freedom. Main effects were followed by Bonferroni-corrected pairwise comparisons. The grand-averaged ERPs at the Fz, FCz, Cz, CPz, and Pz electrode sites for high- and low-CSE athletes under the feedback stage are shown in Figure 3.

## Results

### Behavioral Data

Statistical analysis was conducted on the average response time of the participants under stress. The average response time of the athletes in the high-CSE group under stress (*M* = 1,472.23 ms, SD = 190.62) was significantly faster than that of the low-CSE group (*M* = 1,613.16 ms, SD = 229.65), with *t*(39) = −2.14, *p* = 0.038, *d* = −0.669. Thus, athletes in the high-CSE group responded more quickly under stress.

Statistical analysis was also conducted on participants’ accuracy rate. The accuracy rates of athletes in the high-CSE group (*M* = 53.85%, SD = 2.87) and the low-CSE group (*M* = 53.02%, SD = 4.32) were not significantly different, with *t*(39) = 0.73, *p* = 0.473. The participants’ accuracy rate also suggested that the difficulty of the arithmetic problems in the experimental materials was appropriate and that the experimental materials were scientifically prepared.

### Electrophysiological Data

For stress response phase, regarding the N1 peak latency, the main effect of the right/left hemisphere was significant [*F*(1, 37) = 6.88, *p* = 0.013, $\eta_{p}^{2}$ = 0.157]. The N1 peak latency in the left hemisphere (*M* = 183.29 ms, SD = 2.35) was significantly longer than the right hemisphere (*M* = 178.69 ms, SD = 2.87). The main effect of the subject groups was significant [*F*(1, 37) = 4.76, *p* = 0.036, $\eta_{p}^{2}$ = 0.114]. The N1 peak latency of the low-CSE group (*M* = 186.39 ms, SD = 3.63) was significantly longer than the high-CSE group (*M* = 175.59 ms, SD = 3.36). For N1 peak latency, there were no other interaction effects. For N1 amplitude, the main effect of the groups was significant [*F*(1, 37) = 6.06, *p* = 0.019, $\eta_{p}^{2}$ = 0.141]. The N1 amplitude of the low-CSE group (*M* = −3.95 μV, SD = 0.68) was significantly larger than the high-CSE group (*M* = −1.68 μV, SD = 0.63). There were no other interaction effects for N1 amplitude.

For P2 amplitude, the main effect of the brain region was significant [*F*(1.4, 74) = 6.79, *p* = 0.007, $\eta_{p}^{2}$ = 0.155]. The P2 amplitude of the frontal region of the brain (*M* = 2.57 μV, SD = 0.55) was significantly greater than the frontal-central zone (*M* = 2.33 μV, SD = 0.50) and the central zone (*M* = 1.86 μV, SD = 0.49). The main effect of the groups was not significant [*F*(1, 37) = 0.60, *p* = 0.442]. There were no other interaction effects for P2 amplitude.

For reaction feedback phase, regarding the FRN amplitude, the main effect of the feedback type was significant [*F*(1, 37) = 42.97, *p* < 0.001, $\eta_{p}^{2}$ = 0.558]. The FRN amplitude under error feedback (*M* = 3.37 μV, SD = 1.11) was significantly larger than the correct feedback (*M* = 9.05 μV, SD = 1.07). There is a significant interaction between groups and feedback type [*F*(1, 37) = 17.25, *p* < 0.001, $\eta_{p}^{2}$ = 0.337]. Simple effects analysis revealed that high-CSE group has significant difference in FRN amplitude on feedback type [*F*(1, 37) = 64.50, *p* < 0.001, $\eta_{p}^{2}$ = 0.655]. The FRN amplitude under error feedback (*M* = −0.32 μV, SD = 1.48) is greater than under the correct feedback (*M* = 8.97 μV, SD = 1.42). Low-CSE group has no significant difference in feedback types. For FRN amplitude, there was a significant difference between the groups in the error feedback [*F*(1, 37) = 11.09, *p* = 0.002, $\eta_{p}^{2}$ = 0.246], and the FRN amplitude of error feedback in high CSE group (*M* = −0.32 μV, SD = 1.48) was significantly larger than the low CSE group (*M* = 7.05 μV, SD = 1.65). For dFRN amplitude, the main effect of the groups was significant [*F*(1, 37) = 10.14, *p* = 0.003, $\eta_{p}^{2}$ = 0.215]. The dFRN amplitude of high CSE group (*M* = −8.71 μV, SD = 1.23) was significantly larger than the low CSE group (*M* = −2.94 μV, SD = 1.33).

For P300 amplitude, the groups × feedback type interaction was significant [*F*(1, 37) = 6.19, *p* = 0.018, $\eta_{p}^{2}$ = 0.154]. Simple effects analysis revealed that high-CSE group has significant difference in P300 amplitude on feedback type [*F*(1, 37) = 5.48, *p* = 0.025, $\eta_{p}^{2}$ = 0.138]. The P300 amplitude of correct feedback (*M* = 10.86 μV, SD = 1.54) is larger than error feedback (*M* = 7.39 μV, SD = 1.62). For P300 amplitude, neither the main effects nor interaction effects was significant.

## Discussion

This study used arithmetic problems with the characteristics of uncontrollability and social-evaluated threat to examine differences between athletes with high and low CSE levels under acute psychological stress. The behavioral results of the reaction time and accuracy rate indicated that among athletes with comparable accuracy rates, the reaction time of athletes in the high-CSE group was significantly faster than that of the athletes in the low-CSE group, and thus, athletes in the high-CSE group had a competitive advantage in terms of response speed, agility, and action speed under acute psychological stress. To further explore the reasons for this advantage, differences in cortical neurological activity under acute psychological stress between high- and low-CSE athletes were analyzed in both the stress reaction and response feedback stages.

Competitive sports are mostly performed under intense time pressure, requiring athletes to perform rapid sensory perception and movement initiation (Hülsdünker et al., 2018). When stressed, the brain must quickly and effectively detect information and re-integrate physiological and psychological resources to effectively cope with the stressful stimuli. Therefore, when athletes are stressed, effective cognitive processing is crucial for optimal performance (Sanchez-Lopez et al., 2016). The N1 component primarily reflects the functional role of stress in regulating early sensory coding (Löw et al., 2015; Qi et al., 2018). The results of this study show that in the state of stress, the N1 amplitude of athletes in the low-CSE group was significantly larger than that of athletes in the high-CSE group, and the peak latency of N1 was significantly longer than that of athletes in the high-CSE group. Amplitude is generally believed to reflect the excitability of the brain, whereas the latency period reflects the speed and evaluation time of neurological activity and processing (Wang et al., 2012; van Dinteren et al., 2014). Previous studies have shown that when the perception load caused by high vigilance is increased, the individual’s recognition and processing of stimuli become difficult, prompting an increase in the amplitude of N1 (Yang et al., 2012; Qi et al., 2018). The study by Wang et al. (2012) showed that older people experience larger N1 amplitudes due to slower perception processing and increased difficulty in identifying the target stimuli. In this study, low-CSE athletes had a poorer self-evaluation of their coping ability. Under acute psychological stress, they were more likely to experience a “blank brain,” resulting in increased vigilance and enhanced sensory input. Consequently, both the difficulty of stimulation recognition and processing and the time required for individual perceptual analysis increased, thus reducing response speed. By contrast, high-CSE athletes displayed the confidence of “keep calm even in face of danger” under stress. Therefore, when facing the same stimulating materials, they exhibited low levels of attention and perception load; hence, they could locate and process the related information more quickly, resulting in improved efficiency in processing information and quicker reaction times compared with low-CSE athletes.

Qi et al. (2017) compared electrophysiological responses under stress and no stress and found that the attention processes and cognitive control were regulated by acute psychological stress, which negatively impacted early perception processes, as evidenced mainly by the reduction of the P2 component. The P2 component was a distinct positive waveform in the prefrontal region that occurs after the N1 component and has a latency period of approximately 200 ms. The P2 component was more specifically a cognitive processing component that influences the early process of decision-making and indicated the choice of attention resources and the early outcome of decision-making (Rigoni et al., 2010). Paynter et al. (2009) showed that the larger the P2 amplitude, the more an individual was inclined to adopt a smooth intuitive heuristic strategy. In the present study, the difference in P2 amplitude between the two groups of athletes was not significant, possibly due to factors such as the number of participants and experimental materials. The psychological stress state in this study was stimulated by time-stressed arithmetic problems, which were not the type of sports problems at which athletes are proficient. Therefore, there was no difference in attention resource selection and decision-making processing strategy. In the future research, motor imagery (MI) task can be considered to study (Cebolla et al., 2015), and the distinguishing sports problems that athletes are good at solving can be used as the experimental materials for analysis.

Athletes’ evaluation of the outcome of stress events directly affects their arousal level and emotional state and determines whether they can recover quickly and adapt to the stressful environment during competition (Anshel and Anderson, 2002). Therefore, this study analyzed the changes in the brain activity of athletes with different CSE levels following feedback from transient stress events. FRN and P300 are the two most common EEG components in outcome evaluation; they characterize, respectively, the early warning and early signaling stages that must be changed and the late stage involving the integration of information from the updated behavioral characterization provided by the neural mechanism of outcome evaluation and behavioral regulation (Wu and Zhou, 2009; Leng and Zhou, 2010). In the early stage of primary automated processing, error feedback triggered a larger FRN amplitude, suggesting that negative feedback can also induce an individual’s stress response (Atchley et al., 2017). FRN originates from the anterior cingulated cortex (ACC), which is a negative waveform that appears in the central part of the forehead approximately 250 ms after the presentation of the feedback stimulus (Miltner et al., 1997; Holroyd and Coles, 2002). Mars et al. (2004) stated that FRN mainly transmitted an early warning signal concerning whether the result was “good” or “bad.” Therefore, FRN is sensitive to correct feedback and error feedback stimuli reflects the rapid and difficult process of evaluating the importance of the stimuli, thus providing information for behavioral adjustment. The results of the current study showed that both the FRN and dFRN amplitudes of athletes in the high-CSE group were significantly larger than those of the low-CSE group. Accordingly, in the early stage of feedback processing, athletes with high-CSE were more alert to the error signal of the response. This type of vigilance is highly adaptive because it conveys early warning signals for adjustment, which is conducive to behavioral adjustment in a stressful environment full of uncertainties and can thereby help athletes avoid repeating mistakes.

The anterior cingulated cortex (ACC) conveys the warning that behavior must be adjusted. This information only indicates the occurrence of an error and the necessity of change. The specific behavior adjustment required should be determined by the integration of all information to ensure that behavioral characterization is appropriately updated. This processing is a slower, more detailed, and more sophisticated form of information processing, which may be reflected in the P300 component in the late stage (Mars et al., 2004; Polich, 2007). As a late control evaluation process based on motivation/emotional meaning or attention resource allocation, P300 reflects the transfer of attentional resources or the update of working memory and is positively correlated with the amount of invested psychological resources (Yu and Sun, 2013). The study by Kopp and Lange (2013) showed that in the cued task-switching paradigm, unexpected signal switching triggered a larger P300 amplitude. Similarly, the study by Chase et al. (2011) showed that the behavioral reversal based on explicit rules induced a greater P300 amplitude than that caused by information without behavioral reversal. These studies suggested that the P300 wave may be an EEG indicator that guided behavioral regulation, possibly because P300 reflected the renewal and adjustment of behavioral characterization. In this study, in the late sophisticated processing stage, the P300 amplitude of high-CSE athletes was significantly larger with correct feedback than with error feedback. By contrast, the P300 amplitude in low-CSE athletes was greater with error feedback than with correct feedback, while the difference was not statistically significant. Consequently, after the approximate and automatic early detection of feedback had provided early warning information indicating the necessity of change, the athletes paid greater attention to the stimulus information and engaged in controlled processing. Athletes in the high-CSE group invested more attention resources to correct feedback during stimulation processing to update behavioral characterization and guide behavioral regulation. This positive sophisticated processing is conducive to maintaining the individual’s coping confidence under stress and encouraging the individual to develop positive coping strategies (Snyder, 1999).

The perception of athletes depends on the interaction of the physical characteristics of the object and the athletes’ ability in the environment (Gray, 2014). Coping self-efficacy as an individual resource can affect coping behavior by regulating cognition, emotion, and inclination (Bandura, 1997). The study on the cerebral cortical nerve activity of athletes with high and low CSE levels under acute psychological stress showed that low-CSE athletes lacked confidence and hence were more likely to not know what to do when facing stress. When the stress feedback results were presented, high-CSE athletes were more alert to the error feedback compared with low-CSE athletes and transmitted an early warning signal indicating the necessity of behavioral adjustment. High-CSE athletes could also recover quickly from frustration and disappointment and focus on positive information. By contrast, low-CSE athletes paid greater attention to negative information and the consequences of failure, not only causing them to lose confidence in their abilities but also affecting their subsequent coping behavior. Therefore, coping effectiveness training (CET) and an attention modification program should be incorporated in the training of athletes (Amir et al., 2009; Reeves et al., 2011). Through measures such as improving athletes’ confidence, suppressing attention to negative stimuli to complete the search for positive stimuli, and changing the attention mode, the individual’s self-efficacy can be improved. Athletes can thus better cope with and eliminate interference caused by competitive pressures and eventually achieve their best performance.

Despite these contributions, some limitations in our work should be noted that may shed light on future research directions. The first concern is the use of mental arithmetic exercise for inducing athletes’ psychological stress response. Although mental arithmetic exercise as an effective approach to induce individual psychological stress response, (Dedovic et al., 2005; Qi et al., 2016), whether there is consistency across different kinds of laboratory-induced stress and whether different types of stress sources can trigger the same pattern of electrophysiological response still need to be further studied and tested. Therefore, motor imagery (MI) can be used for psychological simulation of sports stress in future research, for MI can be applied to event-related potential technologies without any interference of real movement (Machado et al., 2013; Cebolla et al., 2015). At the same time, the time-frequency measurement of ERP has been reliably applied to MI (Machado et al., 2013; Tabrizi et al., 2013), and the time-frequency analysis with time-frequency characteristics of EEG oscillations can better reveal brain function activities of athletes under stressful scenarios. Second, another limitation of our study is that we did not measure the objective physiological indicators of psychological stress. In future studies, research findings can be more convincing by increasing the measurement frequency of heart rate, saliva cortisol and other objective stress indicators, raising the sample size, and establishing a correlation test between ERP components and physiological data, which makes the results more convincing.

## Conclusion

The results of the current study show that, under the acute psychological stress, the athletes with low CSE have higher level of vigilance and sensory input which affects the speed of recognition and processing of stimulation and increases the time required for individual perceptual analysis. It eventually will show a decrease in the rate of response. However, in the process of stress response evaluation, athletes with high CSE are more alert to the wrong signal of the result and have adaptive significance in the early feedback result processing stage of providing early warning information. And in the late sophisticated processing stage of affect behavior adjustment, athletes with high and low CSE showed obvious mood congruent effect.

## Ethics Statement

All procedures performed in studies involving human participants were in accordance with the ethical standards of the institutional and/or national research committee and with the 1964 Helsinki Declaration and its later amendments or comparable ethical standards with written informed consent from all subjects. This research was approved by the Human Research Ethics Committee (HREC) at Henan University.

## Author Contributions

YN contributed to developing the theoretical framework, data analysis, organization, and overall writing of the paper. QL contributed to the editing and organization of the paper as well as the overall design. TG contributed to the design, data analysis, and editing of the paper. HH was concerned with drafting the work and revising it critically.

### Conflict of Interest Statement

The authors declare that the research was conducted in the absence of any commercial or financial relationships that could be construed as a potential conflict of interest.
